# MR Assessment of Acute Pathologic Process after Myocardial Infarction in a Permanent Ligation Mouse Model: Role of Magnetic Nanoparticle-Contrasted MRI

**DOI:** 10.1155/2017/2870802

**Published:** 2017-10-18

**Authors:** Cheongsoo Park, Eun-Hye Park, Jongeun Kang, Javeria Zaheer, Hee Gu Lee, Chul-Ho Lee, Kiyuk Chang, Kwan Soo Hong

**Affiliations:** ^1^Bio-Imaging Research Team, Korea Basic Science Institute, 161 Yeongudanji-ro, Ochang-eup, Cheongwon-gu, Cheongju 28119, Republic of Korea; ^2^Cardiovascular Center and Division of Cardiovascular Medicine, Seoul St. Mary's Hospital and College of Medicine, The Catholic University of Korea, 222 Banpo-daero, Seocho-gu, Seoul 06591, Republic of Korea; ^3^Graduate School of Analytical Science and Technology, Chungnam National University, 99 Daehak-ro, Yuseong-gu, Daejeon 34134, Republic of Korea; ^4^Immunotherapy Convergence Research Center, Korea Research Institute of Bioscience and Biotechnology, 125 Gwahak-ro, Yuseong-gu, Daejeon 34141, Republic of Korea; ^5^Department of Biomolecular Science, University of Science and Technology (UST), 217 Gajeong-ro, Yuseong-gu, Daejeon 34113, Republic of Korea; ^6^Laboratory Animal Center, Korea Research Institute of Bioscience and Biotechnology, 125 Gwahak-ro, Yuseong-gu, Daejeon 34141, Republic of Korea

## Abstract

We evaluated the relationship between myocardial infarct size and inflammatory response using cardiac magnetic resonance imaging (CMR) in an acute myocardial infarction (AMI) mouse model. Myocardial infarction (MI) was induced in 14 mice by permanent ligation of the left anterior descending artery. Late gadolinium enhancement (LGE), manganese-enhanced MRI (MEMRI), and magnetofluorescent nanoparticle MRI (MNP-MRI) were performed 1, 2, and 3 days after MI, respectively. The size of the enhanced lesion was quantitatively determined using Otsu's thresholding method in area-based and sector-based approaches and was compared statistically. Linear correlation between the enhanced lesion sizes was evaluated by Pearson's correlation coefficients. Differences were compared using Bland-Altman analysis. The size of the inflammatory area determined by MNP-MRI (57.1 ± 10.1%) was significantly larger than that of the infarct area measured by LGE (40.8 ± 11.7%, *P* < 0.0001) and MEMRI (44.1 ± 14.9%, *P* < 0.0001). There were significant correlations between the sizes of the infarct and inflammatory lesions (MNP-MRI versus LGE: *r* = 0.3418, *P* = 0.0099; MNP-MRI versus MEMRI: *r* = 0.4764, *P* = 0.0002). MNP-MRI provides information about inflammatory responses in a mouse model of AMI. Thus, MNP-MRI associated with LGE and MEMRI may play an important role in monitoring the disease progression in MI.

## 1. Introduction

Although there have been noteworthy improvements in the treatment of patients with myocardial infarction (MI), the incidence of heart failure after MI continues to be high and survival in patients with heart failure following MI remains poor [1, 2]. Myocardial inflammation in response to necrotic tissue generally follows as early as 30 min after the AMI, whereas the number of macrophages peaks around day 3 [3–5]. Macrophages initially accumulate in the infarct border zone adjacent to the ischemic tissue to clear necrotic cellular debris [6].

However, improper or excessive inflammatory responses cause irreversible loss of myocardial function due to inadequate cellular repair processes, tissue injury, and dysfunction of the left ventricle (LV) [7]. In recent years, as our understanding of the biology and physiology of inflammation has improved, the oversimplified model used to describe the process of inflammation in the past has been reconsidered [8]. To ensure favorable scar formation in the infarcted tissue and to ameliorate adverse remodeling, appropriate regulation of the inflammatory response is increasingly considered to be important [9]. Since the cardiac inflammatory response during the progression of MI is a critical therapeutic target, estimation of the relationship between myocardial infarction and the subsequent infiltration of immune cells is important for understanding the process of inflammation in response to myocardial damage.

Several studies have visualized inflammatory response early after MI, including studies using MRI with iron-oxide-based nano-/microparticles and fluorine/gadolinium-containing nanoemulsion, as well as positron-emission tomography (PET) with ^18^F-fluorodeoxyglucose (FDG) [10–12]. FDG allows imaging of inflammation, as it avidly accumulates in macrophages that are metabolically active in the inflammatory phase [13]. However, because of the presence of enhanced FDG uptake in regions with viable myocytes, use of FDG for imaging of inflammation is not straightforward [14].

Several different types of nanoparticles have been evaluated as agents for assessment of myocardial inflammation via MR imaging; these include iron-oxide nanoparticles and fluorine-loaded nanoemulsion [15, 16]. F-loaded nanoparticles were used to monitor the healing process after MI [17]. Micrometer-sized iron oxide particles were injected intravenously before MI induction to efficiently label inflammatory cell for MRI-based cell tracking* in vivo* [18]. Higher phagocytic uptake of magnetofluorescence during the early inflammatory response can be employed to image the infiltrated region in the inflamed myocardium of experimental autoimmune myocarditis [19].

We hypothesized that there is a significant correlation between the size of the infarct and inflammatory lesions. Accordingly, the aim of our study was to assess the relationship between the infarct size and the subsequent inflammatory response using contrast-enhanced cardiac MRI (CMR) in a mouse model of AMI. Myocardial infarction size was evaluated using late gadolinium enhancement (LGE) and manganese-enhanced MRI (MEMRI), and subsequently, the inflammatory lesion size was assessed by MRI using magnetofluorescent nanoparticles (MNPs). Moreover, the size of the contrast-enhanced lesion in CMR was calculated using both area-based and sector-based approaches, to reduce the effect of geometrical alterations in LV myocardium during the progression of the MI [20, 21].

## 2. Materials and Methods

### 2.1. Acute Myocardial Infarction Model

All animal procedures were approved by the Institutional Animal Care and Use Committee of Korea Basic Science Institute (KBSI-AEC 1510). Fourteen male C57BL/6 mice ranging from 10 to 14 weeks of age were used in this study. Mice were anesthetized by inhalation of a mixture of isoflurane (1.5%) and oxygen. After anesthesia, mice were intubated with an endotracheal catheter and ventilated using a rodent ventilator (Harvard Apparatus, Inc., Holliston, MA, USA) and were placed on a temperature controlled heating pad. A left thoracotomy was performed through the fourth intercostal space. The pericardial sac was opened to access the heart, and permanent ligation of the anterior descending branch of the left coronary artery was achieved by tying an 8-0 nylon suture around the artery. Myocardial ischemia was confirmed by observing blanching and dyskinesia of the anteroapical region of the LV distal to the suture.

### 2.2. Cardiac MRI Protocols

All CMR experiments were performed on a 4.7 T animal MRI system (Biospec 47/40, Bruker BioSpin, Ettlingen, Germany). The radiofrequency coil for transmitting pulses and receiving signals was a 35 mm diameter birdcage resonator. The body temperature of the mice was maintained at ca. 37°C using a warm air blower. Electrocardiography (ECG) electrodes were inserted into the fore- and hind-limbs and a respiration pillow was taped across the chest. ECG and respiration signals were monitored using a small animal monitoring unit (SA Instruments, Inc., Stony Brook, NY, USA). Short-axis CMR image acquisition was both ECG- and respiratory-gated.

One day after MI induction, LGE was acquired 30 min after intravenous injection of Gd-DTPA-BMA (0.3 mmol/kg, Omniscan®, GE Healthcare, Pittsburgh, PA, USA). At 2 days after coronary occlusion, MEMRI was obtained 1 h after an intravenous infusion of MnCl_2_ (0.2 mmol/kg; Sigma-Aldrich, St Louis, MO, USA) using an infusion pump at a flow rate of 0.5 *µ*L/min, in order to prevent cardiotoxic side effects [22]. A 24 h interval between LGE and MEMRI was allowed to avoid interference of the enhancement [23].

For both LGE and MEMRI imaging, a T_1_-weighted FLASH sequence was used and parameters were as follows: TR/TE = 73/2.7 ms, flip angle = 60°, field of view = 30 × 30 mm^2^, matrix size = 256 × 256, and slice thickness = 1 mm. Immediately after the MEMRI scan, MNPs were given intravenously to the same mice, as previously described (a detailed description of nanoparticles can be found in Supplementary Material available online at https://doi.org/10.1155/2017/2870802) [19], and MNP-MRI was performed 24 h after injection of MNPs (10 mg Fe/kg) by using a T_2_^*∗*^-weighted FLASH sequence with the following imaging parameters: TR/TE = R-R interval/6 ms, flip angle = 30°.

### 2.3. Histological Examination

After all MRI studies, mice were sacrificed and hearts were extracted. Heart samples were thoroughly washed in saline solution and embedded in optimal cutting temperature (OCT) compound (Sakura Finetek, Torrance, CA, USA) and cut into 5 *μ*m thick short-axis slices. To evaluate the necrotic myocardium, immunohistochemistry was performed on frozen tissue sections using a primary antibody against myoglobin (ab77232, Abcam, Cambridge, MA, USA) [24, 25]. To detect necrotic myocardium, a HistoMouse™-MAX kit (Invitrogen, Waltham, MA, USA) was used to immunostain the sections, and color was developed using the Fast DAB with metal enhancer tablet set (Sigma-Aldrich). To analyze macrophage distribution, heart sections were stained with a primary antibody against CD68 (ab53444, Abcam) and Alexa Fluor 488-conjugated secondary antibodies (Molecular Probes, Eugene, OR, USA). The sections were counterstained with DAPI to visualize the nuclei. We evaluated colocalization of MNPs and macrophages in inflamed myocardium by color-coding the confocal laser scanning microscopic images using red for RITC (MNP), green for Alexa Fluor488 (macrophages), and blue for DAPI (nuclei). Stained tissue sections were digitally scanned using a slide scanner (SCN400, Leica, Oberkochen, Germany).

### 2.4. Image Processing and Analysis

All MRI images were analyzed using both area-based and sector-based approaches by means of ImageJ (National Institutes of Health, Bethesda, MD, USA) and Segment (Medviso, Lund, Sweden), respectively. To evaluate the size of lesions showing hyperenhancement (LGE) or hypoenhancement (MEMRI and MNP-MRI), LV myocardial borders were delineated manually and the sizes of enhanced lesions on LGE, MEMRI, and MNP-MRI were quantified using Otsu's thresholding method. Otsu's method calculates the threshold value from a histogram of signal intensity to acquire minimal variance both above and below the threshold. Angular sector values were acquired from the region of the LV with an angular resolution of 1°. Enhancement size, expressed as a percentage, was calculated by dividing the enhanced lesion size by the LV size for each of the 4 slices below the mid-ventricular level.

### 2.5. Statistical Analysis

All data are expressed as mean ± SD. Statistical analysis of enhanced lesion sizes was performed using Prism (GraphPad Prism v5.0, San Diego, CA, USA). After using the Shapiro-Wilk normality test, repeated-measures one-way ANOVA and Tukey's multiple comparisons test were used for statistical analysis of enhanced sizes. Linear trends were evaluated using linear regression analysis. Linear correlation between enhanced lesion sizes was evaluated using Pearson's or Spearman's correlation coefficients. Enhanced lesion sizes between the area-based and sector-based approaches were compared using Bland-Altman analysis. *P* < 0.05 was considered statistically significant.

## 3. Results

**Figure 1:**
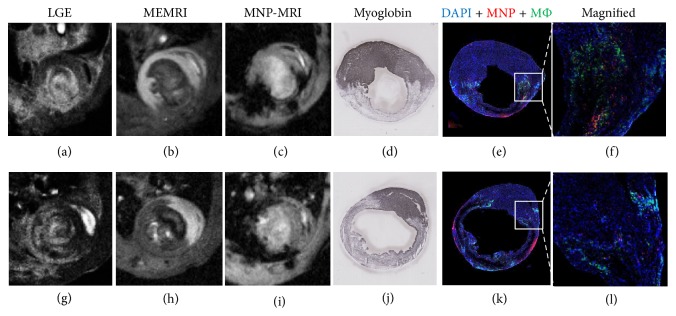
MRI images were acquired at 1 day (LGE: a, g), 2 days (MEMRI: b, h), and 3 days (MNP-MRI: c, i) after induction of myocardial infarction (MI). Histological examination of the mouse heart was performed at 3 days after MI (d, j). Representative cardiac MRI images and corresponding histological images are shown. Images in the upper row were acquired from anterolateral LV wall infarction (a–f) and bottom row images were obtained from entire LV free wall infarction (g–l). The infarcted myocardium appears hyperintense in LGE and hypointense in MEMRI images, and the corresponding infarct region is evident in a immunohistochemistry stain for myoglobin (d, j); myoglobin-positive areas (dark blue) are viable myocardium. MNP- (red) and CD68-positive (green) regions are consistent with hypointense signals in MNP-MRI representing inflammatory areas after myocardial infarction (e, k). Colocalization of MNPs with macrophages is observed in the peri-infarct zone (f, l).

Representative enhanced CMR images with 3 contrast agents and histological results from 2 different mice are shown in Figure 1. Hyperintense lesions on LGE images and hypointense lesions on MEMRI images revealed that the infarct area corresponded with the myoglobin-negative region on immunohistochemistry images. Inflammatory regions in the same animal were hypoenhanced on MNP-MRI images and were confirmed by immunohistochemistry staining of macrophages that colocalized well with fluorescent MNP distribution. No nuclei stained with DAPI were found within the necrotic core at 3 days after MI. Macrophages initially accumulated in the infarct border zone.

**Figure 2:**
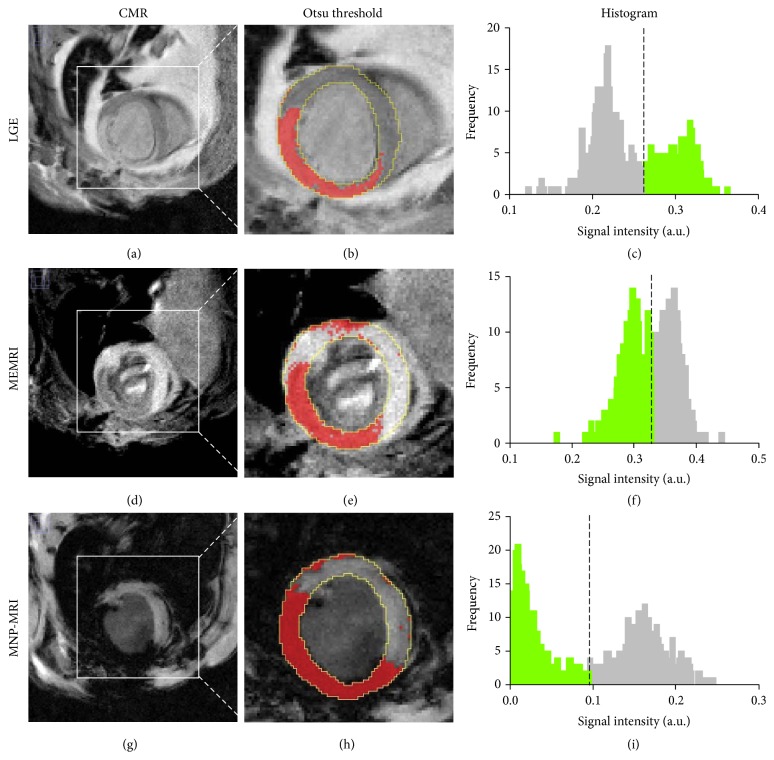
Area-based approach for contrast-enhanced size measurement. Enhanced area (red color) was demarcated using Otsu's threshold method (b, e, h). The epicardium and endocardium are traced in yellow. The dotted lines in the histogram correspond to the estimated threshold values and green bars represent enhanced pixels in MRI images (c, f, i). The sizes of enhanced area for LGE, MEMRI, and MNP-MRI was 38.7%, 46.3%, and 50.4%, respectively. The horizontal axes represent normalized signal intensity in the MR image.

Hyper- and hypoenhanced areas were segmented using Otsu's threshold method in both area-based and sector-based approaches.  Figure 2 shows Otsu's thresholding results delineating hyper- and hypoenhanced regions of the LV on contrasted CMR images using the area-based approach. Threshold values are shown in histograms of normalized signal intensity distribution. For this case, threshold values for LGE, MEMRI, and MNP-MRI were 0.26, 0.33, and 0.10, respectively.

**Figure 3:**
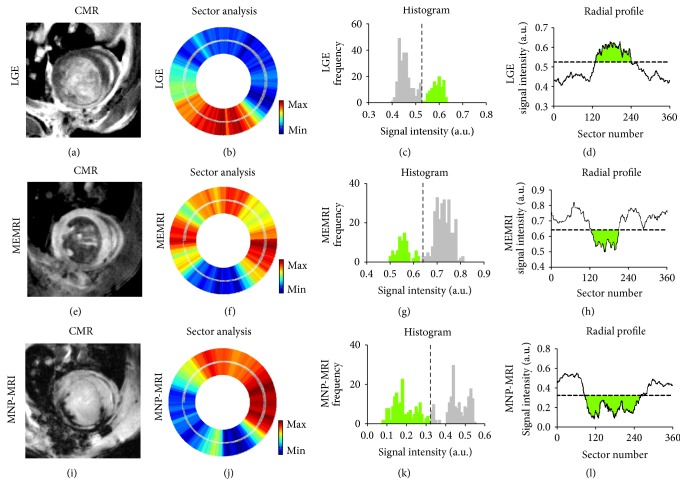
Sector-based analysis of contrasted CMR images. LV myocardium was registered to 360 radial segments for sector analysis (b, f, j). Enhanced sectors were determined by Otsu's thresholding (c, g, k). The green region denotes the enhanced area both in the histogram and in radial profile. Dashed lines both in the histogram and in radial profile are threshold values according to Otsu's method. The enhanced sizes as determined by LGE, MEMRI, and MNP-MRI were 30.8%, 23.9%, and 49.2%, respectively.

In the sector-based method (Figure 3), analysis of signal intensity was performed along 360 radial sectors around the LV. Threshold values determined by Otsu's method are denoted both in the histograms and in the corresponding radial profiles. The segmental distribution of signal intensity is presented in radial profiles. The threshold values for LGE, MEMRI, and MNP-MRI for this case were 0.53, 0.64, and 0.32, respectively.

**Figure 4:**
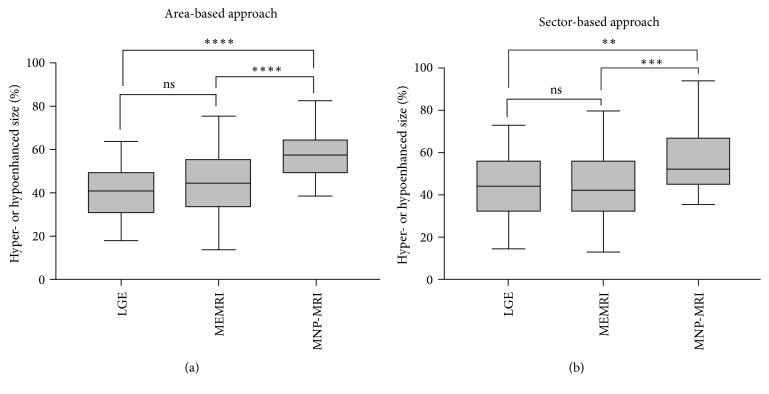
Comparison of contrasted sizes of MI mice by different MRI methods. The sizes were evaluated by area-based (a) and sector-based approaches (b). Enhancement size of MNP-MRI was significantly larger than that of LGE and MEMRI for both approaches. Difference in the enhancement size between LGE and MEMRI was not significant for both approaches. ^*∗∗*^*P* < 0.01, ^*∗∗∗*^*P* < 0.001, ^*∗∗∗∗*^*P* < 0.0001, ns = nonsignificant. (area-based: *P* = 0.34 for LGE versus MEMRI, *P* < 0.0001 for LGE versus MNP-MRI and *P* < 0.0001 for MEMRI versus MNP-MRI; sector-based: *P* = 0.99 for LGE versus MEMRI, *P* = 0.0015 for LGE versus MNP-MRI and *P* = 0.0001 for MEMRI versus MNP-MRI).

There was no significant difference in the infarct lesion size between LGE and MEMRI (Figure 4, area-based: 40.8 ± 11.7% versus 44.1 ± 14.9%, *P* = 0.34; sector-based: 45.0 ± 14.2% versus 44.7 ± 16.6%, *P* = 0.99). Moreover, the area-based and sector-based measurements based on MNP-MRI produced similar results (57.1 ± 10.1% versus 55.7 ± 13.4%, *P* = 0.7365). Bland-Altman analysis (Figure  S1) revealed a good agreement for the infarct lesion size between LGE and MEMRI, with only small differences (−3.3% and 0.4% in the area-based and sector-based approaches, resp.). The extent of the inflammatory lesion determined by the MNP-MRI (area-based: 57.1 ± 10.1%; sector-based: 55.7 ± 13.4%) approach was significantly larger than that determined using LGE (area-based: *P* < 0.0001; sector-based: *P* = 0.0015) and MEMRI (area-based: *P* < 0.0001; sector-based: *P* = 0.001).

**Figure 5:**
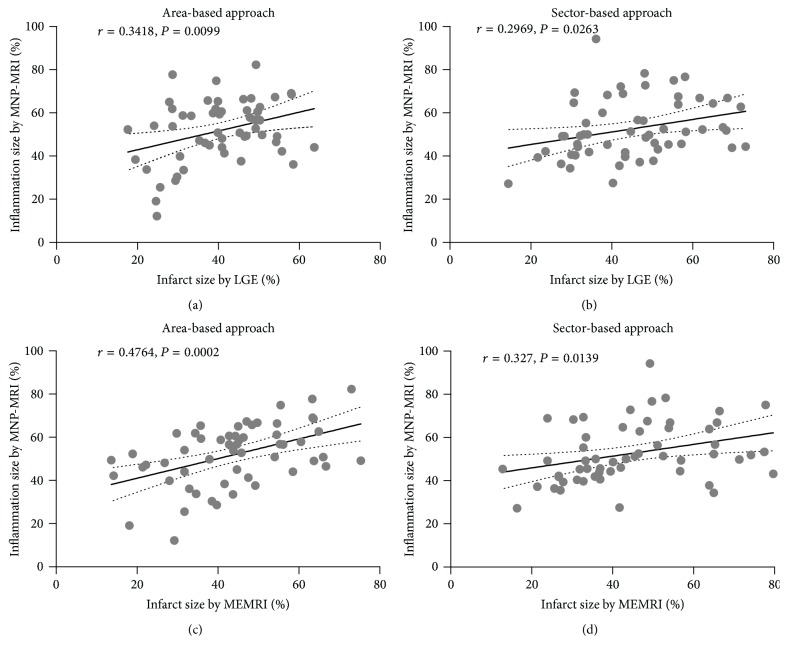
Correlation between infarct lesion size based on LGE or MEMRI and inflammatory lesion size based on MNP-MRI. Pearson's correlation coefficients (*r*) and *P* values are described in the figure. Significant positive correlations between the infarct and inflammatory lesion size were observed by both area-based (a, c) and sector-based approaches (b, d). The solid line denotes the linear regression fit, and the dotted lines represent the 95% confidence limits. Each data point represents the individual enhanced size for each slice (4 slices per mouse).

Furthermore, linear regression analyses revealed a significant correlation between infarct lesion sizes as determined by LGE and MEMRI and the inflammatory lesion size as determined by MNP-MRI (Figure 5). The infarct lesion size determined by LGE correlated positively with the inflammatory lesion size determined by MNP-MRI, for both the area-based (*r* = 0.3418, *P* = 0.0099) and sector-based approaches (*r* = .2969, *P* = 0.0263). Inflammatory lesion size based on MNP-MRI also correlated significantly with infarct lesion size based on MEMRI (area-based: *r* = 0.4764, *P* = 0.0002; sector-based: *r* = 0.327, *P* = 0.0139). Bland-Altman plots (Figure  S2) showed the mean differences between infarct lesion size and inflammatory lesion size as assessed by area-based and sector-based analysis for 3 enhanced CMR images.

**Figure 6:**
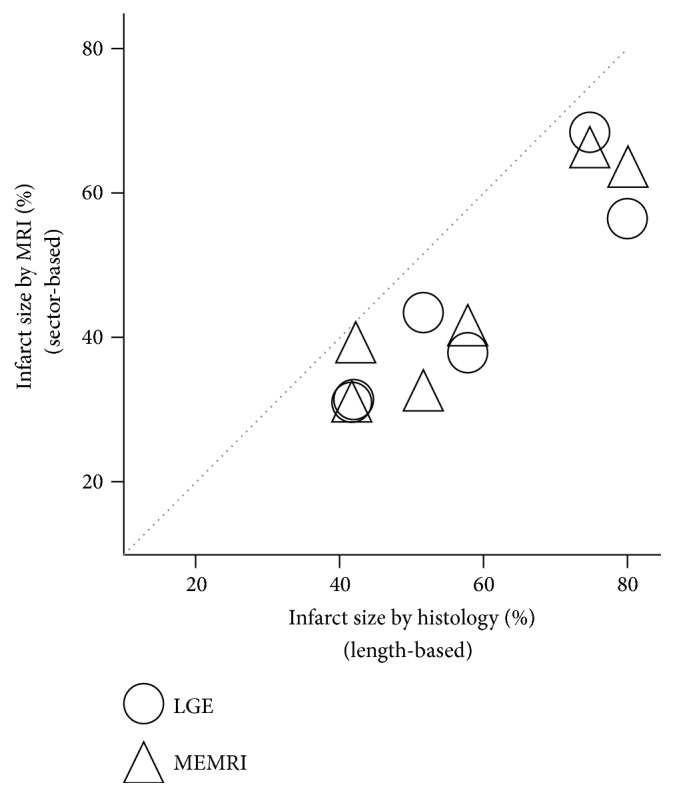
Scatterplot of infarction lesion size based on MRI (sector-based method) and histology (length-based method). There is a significant correlation of infarct size between MRI and histology results. Spearman's rank correlation coefficients (*r*) and *P* values are *r* = 0.9058 (*P* = 0.0129) for LGE (◯), and *r* = 0.9301 (*P* = 0.0071) for MEMRI (△). The dotted line represents the identity line. The infarct size was measured at the mid-ventricular level in 6 mice.

Figure 6 shows the correlation between the extent of infarcts based on MRI and histology, which were measured by sector-based and length-based calculations, respectively. Significant correlations were shown between infarct lesion sizes assessed by MRI and those determined by histology (Spearman's rank correlation coefficient *r* = 0.8857, *P* = 0.0333 for both LGE and MEMRI). Additionally, infarct sizes derived from histological area measurement were significantly correlated with infarct lesion sizes based on measurements made on MRI (Figure  S3, Spearman's rank correlation coefficient *r* = 0.8697, *P* = 0.0333 for LGE and *r* = 0.8407, *P* = 0.0444 for MEMRI).

## 4. Discussion

In our study, during 3 consecutive days of imaging of AMI model mice, the enhanced lesion size was calculated using Otsu's thresholding method. The resultant histogram of signal intensity in the myocardial region showed a bimodal enhanced and nonenhanced distribution. We showed that the inflammatory lesion size on MNP-MRI images showed a statistically significant positive correlation with infarct lesion sizes on both LGE and MEMRI images, using area-based and sector-based approaches. However, all of the correlation degrees were moderate or weak. Furthermore, we compared the size differences among contrast-enhanced cardiac MR images to determine whether the size of the inflammatory lesion was larger than that of the infarct lesion. In the present study, we found that inflammatory and infarct lesion sizes in the acute phase showed comparable results. This therefore confirmed our hypothesis that the extent of the inflammatory response is proportional to the size of the MI in the acute phase.

Our results are consistent with the findings of a prior ^18^F-FDG-PET/MRI study that demonstrated that the extent of ^18^F-FDG uptake correlated with the extent of lesions on LGE (*r* = 0.78, *P* < 0.0001) and significantly exceeded the extent of LGE lesions (33.2 ± 16.2% versus 20.4 ± 10.6%, *P* < 0.0001) [12]. Both the LGE and the extent of ^18^F-FDG uptake also correlated with the peak counts of leukocytes from peripheral blood, CCR2^+^ monocytes, and CD14^high^/CD16^+^ monocytes during the first 3 days after infarction. Thus, monocyte/macrophage release and migration to the heart may depend on the infarct size. Moreover, using Otsu's thresholding technique for assessing the size of the contrast-enhanced lesions on CMR images was observer-independent, reduced bias and variability, and showed a relatively good performance, if the distribution of signal intensity in the region of interest can be assumed to follow a bimodal distribution, such as in LGE, MEMRI, and MNP-MRI images [26]. Our research confirms the findings of Bönner et al. using ^19^F MRI [17]. The major disadvantages of ^19^F MRI are the low spatial resolution, long scan time, and additional requirements for the detection of fluorine signal. In contrast to ^19^F MRI, inflammation imaging with iron-oxide nanoparticle can acquire high spatial resolution images with a short MRI scan time, without requiring fluorine signal detection.

A clinical trial for comparing the inflammatory response assessed by the number of white blood cells (WBC) with the infarct lesion size on LGE images showed a stronger correlation between the WBC number and the infarct size [27]. Accordingly, infarct size measured by LGE affects inflammatory responses during STEMI. Moreover, infarct size determined by transthoracic echocardiography correlated strongly with the number of circulating monocytes in mice [28]. That study also observed a positive correlation between the extent of myocardial injury measured by LGE and the peripheral blood monocyte count in humans. Yang et al. demonstrated a positive correlation between the contrast-to-noise ratio (CNR) in the injured myocardium and the attenuation of left ventricular function after MI, by using micrometer-sized iron-oxide particle-enhanced MRI [18]. Although they described a noninvasive and temporal approach to imaging inflammatory cell recruitment into the infarcted area, they did not measure infarct size with LGE.

We found no significant differences between the 2 geometrical approaches, area-based and sector-based methods, used for calculation of enhanced lesion size from contrast-enhanced cardiac images. The sector-based method follows a similar strategy to the angle-based approach for estimating the enhanced lesion size [20, 21]. In contrast to previous studies on chronic MI, we showed that enhanced lesion size was not significantly different between the area-based and sector-based techniques, probably because morphological alterations are less obvious in the AMI phase.

In our study, infarct lesion sizes determined by LGE and MEMRI were smaller than those determined using immunohistological staining with antimyoglobin antibody. This result differed from the findings of a recent study that compared the sizes of the infarcts measured by MRI and by histological examination [29]. They showed that the infarct lesion sizes measured on postoperative day 7 after MI induction, with LGE, at 7.5 and 10 min after injection for Gd-DTPA, were not significantly different from those determined by ex vivo triphenyltetrazolium chloride (TTC) staining. The CNR peaked at 10 min, after which it decreased gradually over a period of 30 min, which allowed demarcation of the infarct region. Possible reasons for the underestimation in our study were the relatively longer delay time for LGE and the use of a permanent ligation model. In a previous LGE delay-time study in an ischemia/reperfusion model, the specific imaging time after Gd-DTPA injection is crucial for determining the infarct lesion size accurately [30]. The enhancement of LGE occurs only in nonviable myocardium as hyperenhanced regions [31]. The permanent ligation model has a markedly smaller salvageable ischemic border zone that presents as hyperenhancement in LGE than seen in the ischemia/reperfusion model [32].

This study had several limitations, including a relatively small sample size and the use of a permanent ligation model. An ischemia/reperfusion MI model could provide more clinically relevant results than those obtained using a permanent occlusion model, since the mechanisms of injury and inflammatory responses differ, depending on whether the myocardium is subjected to transient or permanent ischemia [33]. Iron-oxide nanoparticles cause a blooming artifact and this should be taken into account when interpreting CMR data for quantifying the inflammatory lesion size in MNP-MRI. This could be reduced by using appropriate imaging parameters allowing for more accurate delineation of the lesion. Furthermore, the difference in scan timing (1 day versus 3 days) is a possible reason for discrepancy between areas of inflammation and necrosis.

## 5. Conclusion

In conclusion, the findings of our study indicate that the combination of LGE, MEMRI, and MNP-MRI can provide information about the inflammatory response to MI in a mouse model of AMI. Histological evaluations confirmed the MRI findings of a clear region of enhancement in necrotic and inflammatory areas. Combination of MNP-MRI with LGE and MEMRI, applied in a mouse model of early stage MI, may play an important role in monitoring the disease progression in MI.

## Supplementary Material

More detailed information on the magnetic nanoparticles used in this study and some supplement results are included in this supplementary material file.
