# Supplementary material for: MR Assessment of Acute Pathologic Process after Myocardial Infarction in a Permanent Ligation Mouse Model: Role of Magnetic Nanoparticle-Contrasted MRI
Source: Contrast Media Mol Imaging. 2017 Oct 18;2017:2870802. doi: 10.1155/2017/2870802 (PMC5664276; doi:10.1155/2017/2870802)
Supplement: Supplementary file 1 — More detailed information on the magnetic nanoparticles used in this study and some supplement results are included in this supplementary material file. [file 2870802.f1.docx]

**MR Assessment of Acute Pathologic Process after Myocardial Infarction in a Permanent Ligation Mouse Model: Role of Magnetic Nanoparticle-Contrasted MRI**

Cheongsoo Park^1^ • Eun-Hye Park^2^ • Jongeun Kang^1,3,4^ • Javeria Zaheer^1,3^ • Hee Gu Lee^4,5^ • Chul-Ho Lee^6^ • Kiyuk Chang^2*^ • Kwan Soo Hong^1,3,4*^

^1^Bio-imaging Research Team, Korea Basic Science Institute, 161 Yeongudanji-ro, Ochang-eup, Cheongwon-gu, Cheongju 28119, South Korea

^2^Cardiovascular Center and Division of Cardiovascular Medicine, Seoul St. Mary’s Hospital and College of Medicine, The Catholic University of Korea, 222 Banpo-daero, Seocho-gu, Seoul 06591, South Korea

^3^Graduate School of Analytical Science and Technology, Chungnam National University, 99 Daehak-ro, Yuseong-gu, Daejeon 34134, Korea

^4^Immunotherapy Convergence Research Center, Korea Research Institute of Bioscience and Biotechnology, 125 Gwahak-ro, Yuseong-gu, Daejeon 34141, South Korea

^5^Department of Biomolecular Science, University of Science and Technology (UST), 217 Gajeong-ro, Yuseong-gu, Daejeon 34113, South Korea

^6^Laboratory Animal Center, Korea Research Institute of Bioscience and Biotechnology, 125 Gwahak-ro, Yuseong-gu, Daejeon 34141, South Korea

**Magneto-Fluorescent Nanoparticles**

Silica- and polyethylene glycol (PEG)-coated superparamagnetic iron oxide (SPIO) nanoparticles were prepared. The nanoparticles had a core size of ca. 12 nm in diameter and the final mean particle size measured by dynamic light scattering was ca. 85 nm, including a rhodamine isothiocyanate (RITC)-incorporated silica coating and a PEG (molecular weight: of 460 - 590; Gelest Inc., Tullytown, PA, USA) layer. The transverse relaxivity (r_2_) at 4.7 T was about 130 s^-1^mM^-1^ and the blood half-life in rats was 2.8 ± 0.5 h. These nanoparticles can be detected by MR and fluorescence imaging.


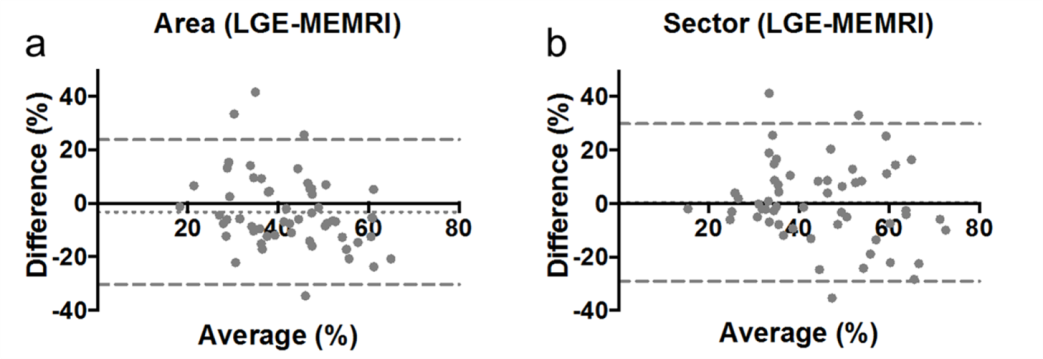


**Figure S1**. Bland-Altman plots displaying 95% limits of agreement between the infarct sizes on LGE and MEMRI images by area- (a) and sector-based (b) methods. (dotted line = mean difference, dashed line = ± 1.96 standard deviations).


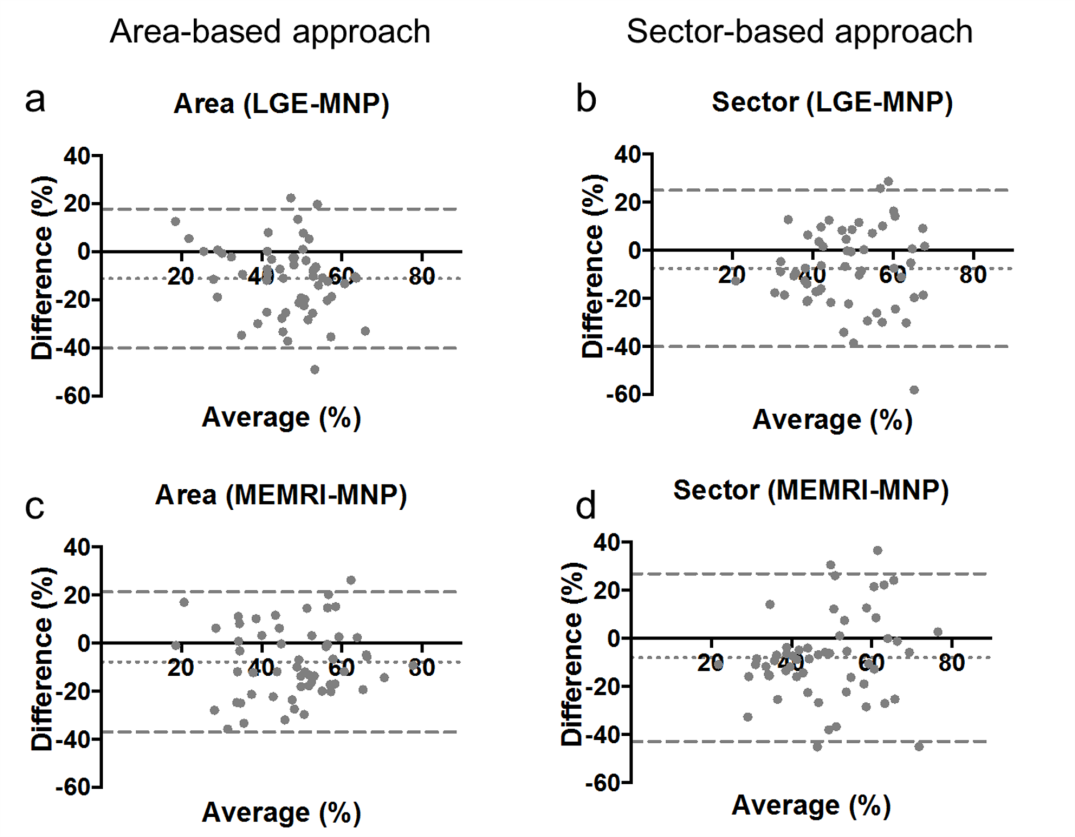


**Figure S2**. Bland-Altman plots for the comparison between infarct size and inflammatory size. Inflammatory lesion sizes from MNP-MRI were larger than infarct lesion sizes from LGE and MEMRI. The mean differences and limits of agreement were −11.2 ± 29.0% (a) and −7.9 ± 29.2% (c) for area-based measurement, and −7.6 ± 32.5% (b) and −8.0 ± 34.9% (d) for sector-based analysis. (dotted line = mean difference, dashed line = ± 1.96 standard deviations).


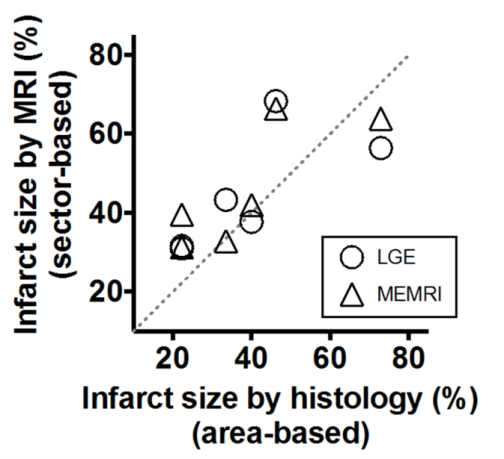


**Figure S3**. Scatterplot of infarction size from MRI (sector-based method) and histology (area-based method). Spearman’s rank correlation coefficients (r) and P values are r = 0.8697 (P = 0.0333) for LGE (○), and r = 0.8407 (P = 0.0444) for MEMRI (△). Dotted line represents identity line. Infarct size was measured at mid-ventricular level in 6 mice.
